# Ultrathin endoscope-assisted endotracheal intubation in the prone position for endoscopic retrograde cholangiopancreatography: a standardized protocol

**DOI:** 10.1055/a-2809-8111

**Published:** 2026-03-09

**Authors:** Yuemin Feng, Yi Liu, Changqin Xu, Maofeng Sun, Tingting Gao, Hongwei Xu, Shulei Zhao

**Affiliations:** 1 34708 Department of Gastroenterology, Shandong Provincial Hospital Affiliated to Shandong First Medical University, Jinan, China; 2 34708 Department of Anesthesiology, Shandong Provincial Hospital Affiliated to Shandong First Medical University, Jinan, China

Endotracheal intubation during endoscopic retrograde cholangiopancreatography (ERCP) enhances patient tolerance, optimizes conditions for prolonged procedures, and reduces complications. Endoscopist-assisted intubation is a rapid and safe technique. Conventional laryngoscopic intubation is limited by standard prone/semi-prone ERCP positioning. While ultrathin endoscopes have been proposed, a standardized protocol is lacking 1 . We describe a standardized technique for ultrathin endoscope-assisted prone intubation prior to ERCP ( Video 1 ).

Ultrathin endoscope-assisted endotracheal intubation in the prone position for ERCP. ERCP, endoscopic retrograde cholangiopancreatography.Video 1

A 68-year-old woman presented with jaundice and pruritus. Laboratory tests showed markedly elevated total bilirubin. Contrast-enhanced computed tomography (CT) suggested obstructive jaundice secondary to suspected cholangiocarcinoma. ERCP was planned for definitive diagnosis and biliary decompression.

**Fig. 1:**
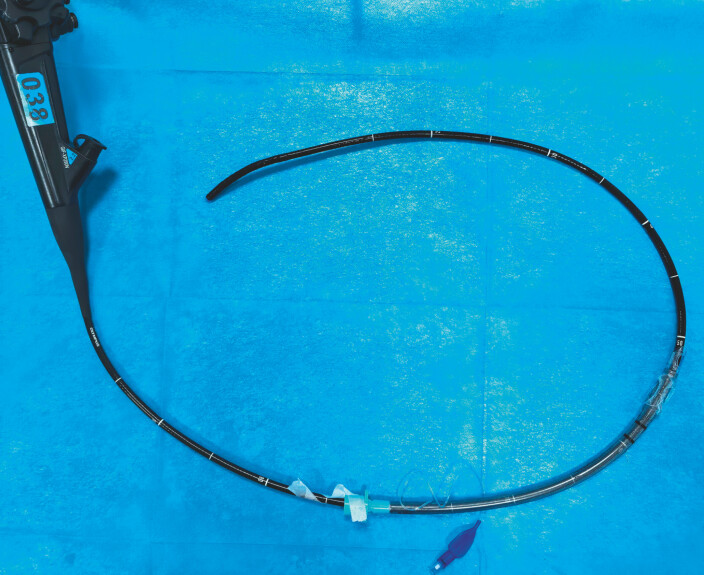
The endotracheal tube is threaded over the lubricated insertion tube of the ultrathin endoscope, with its tip positioned approximately 50 cm from the distal end of the endoscope.

**Fig. 2:**
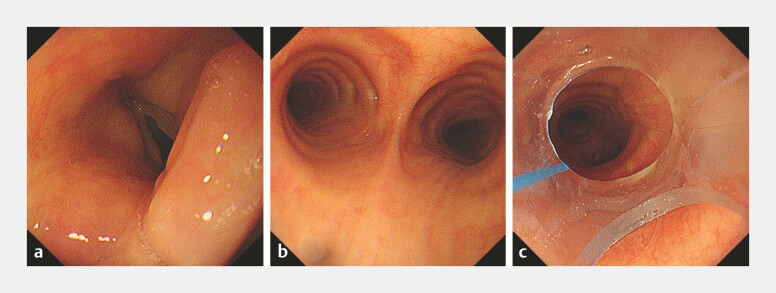
The procedure of ultrathin endoscope-assisted endotracheal intubation: **a** The ultrathin endoscope is advanced through the glottis into the trachea. **b** The ultrathin endoscope is advanced under direct vision to the carina; **c** The tip position of the endotracheal tube is confirmed using the ultrathin endoscope.

A standard endotracheal tube (ETT) is lubricated and pre-threaded over the insertion tube of an ultrathin endoscope, positioning the ETT tip approximately 50 cm proximal to the endoscope distal tip ( Fig. 1 ). The patient is positioned prone for ERCP with head turned right and a bite block in place. Following pre‑oxygenation, general anesthesia is induced using etomidate, sufentanil, and cisatracurium. Positive‑pressure ventilation is administered via a mask until adequate muscle relaxation is achieved. Under direct visualization, the endoscopist (standing on the patient’s right) inserts a lubricated ultrathin endoscope through the side port of the bite block ( Fig. 2 **a** ). The scope is advanced through the oropharynx, passed across the vocal cords, and positioned near the carina ( Fig. 2 **b** ). The anesthesiologist (at head of bed) then threads the pre‑loaded ETT into the trachea. Withdrawing the endoscope, ETT placement is visually confirmed 2–3 cm above the carina ( Fig. 2 **c** ). The cuff is inflated, the tube secured, and the circuit connected to the anesthesia machine. Anesthesia is maintained with propofol and remifentanil infusions.

The patient was successfully intubated using this technique. General anesthesia was maintained effectively throughout the procedure. ERCP with stone extraction was completed uneventfully. The patient was extubated smoothly after the procedure and had an uneventful recovery with no anesthesia- or intubation-related complications during the postoperative follow-up.

**Fig. 3:**
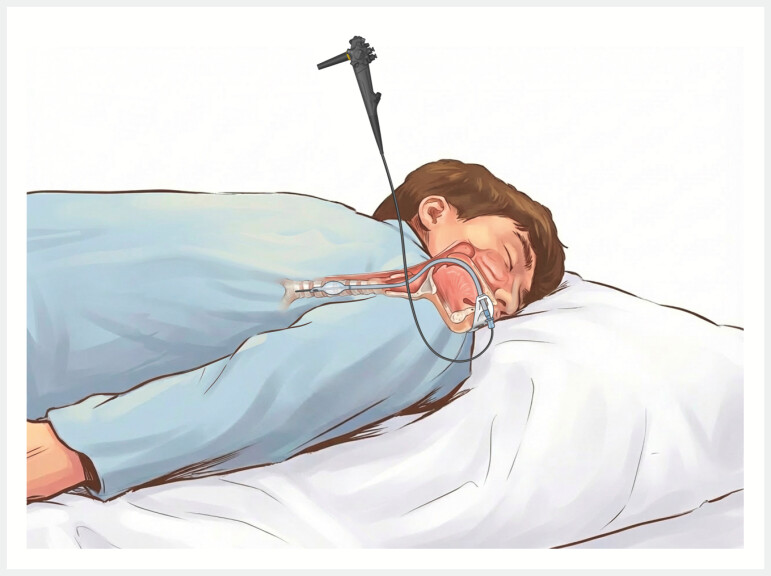
Diagram: ultrathin endoscope-assisted endotracheal intubation.

This technique offers significant benefits. It enables definitive airway establishment in the final posture for ERCP, eliminating risks carried by transferring and the position of the patient ( Fig. 3 ). The fully visualized approach minimizes oropharyngeal trauma and ensures precise ETT placement, significantly reducing intubation time compared to standard intubation. The insertion and confirmation of the endotracheal tube depend on the seamless coordination between the endoscopist and the anesthesiologist. Additionally, the use of an extra endoscope may result in increased decontamination costs for an ultrathin endoscope. It should also be noted that inserting an ultrathin endoscope into the trachea may increase endoscopy-related risks, such as aspiration pneumonia, a concern that requires confirmation through large-scale clinical trials. During the procedure, a strict aseptic technique should be maintained.

Endoscopy_UCTN_Code_TTT_1AR_2AB
